# Supplementary material for: Spatiotemporal patterns of water and vegetation in Poyang Lake from 2013 to 2021 using remote sensing data
Source: PLoS One. 2025 Jul 31;20(7):e0327579. doi: 10.1371/journal.pone.0327579 (PMC12312891; doi:10.1371/journal.pone.0327579)
Supplement: S1 Data — (DOCX) [file pone.0327579.s001.docx]

S1 **Fig 1. Study region of Poyang Lake.**This figure shows the location of Poyang Lake in China and Jiangxi Province, as well as surrounding rivers and elevation information. The map in the upper left corner marks the location of Jiangxi Province (red area) within China. The map in the lower left corner shows the location of Poyang Lake (red rectangular area) within Jiangxi Province, accompanied by an elevation legend (high: 2,147 meters, low: 0 meters). The map on the right shows Poyang Lake and its surrounding rivers (Yangtze River, Xin River, Rao River, Gan River, Fu River, Chang River, Le'an River), along with an elevation legend (high: 2,147 meters, low: 0 meters), scale, and latitude/longitude coordinates.

S1**Fig 3. Spectral reflectance curves of typical land cover types.** Curves showing reflectivity (%) across wavelength (0.2 - 2.0 μm) for various surface types. Water bodies (dashed line), Cities (dashed - dotted line), Soil (solid line), Vegetation (another solid line). Reflectivity varies with wavelength for each type, indicating distinct spectral characteristics.

S1 **Fig 4. Spectral reflectance curves of water bodies with different sediment concentrations.** High resolution curves showing reflectivity values across a specific wavelength range for various water bodies. Lake water (sediment content 47.9 mg/L), Yangtze River water (sediment content 92.5 mg/L), Yellow River water (sediment content 960 mg/L). Each water body's reflectivity varies with wavelength, indicating distinct optical properties based on sediment content differences.

S1 **Fig 6. Selected supervised classification results from 2013 to 2021.** High - resolution maps showing the distribution of different surface types (Water, Vegetation, Mud, Sand) at various time points from 2013 to 2021. Each map corresponds to a specific date, with the legend indicating the color - coding for Water (blue), Vegetation (green), Mud (orange), and Sand (yellow). These maps illustrate the dynamic changes in the lake basin's surface composition over time.

S1 **Fig 7. The inter-annual variation characteristics of land cover areas changes.**(a) Line chart showing the dynamic changes in the area (km²) of Water, Vegetation, and Mud and Sand from 2013 - 05 - 06 to 2024 - 04 - 18. (b) Stacked bar chart presenting the annual proportion (%) of Water, Vegetation, and Mud and Sand from 2013 to 2024. These visualizations illustrate the changing patterns of different surface types over time in the lake basin.

S1 **Fig 8.Intercorrelation between the areas of water,vegetation and sediment.**(a)Scatter plot showing the correlation between vegetation area (y - axis, km²) and water area (x - axis, km²). Each black dot represents a data point reflecting the paired values of vegetation area , water area and mud and sand area.

S1 **Fig 9.The intra-annual variation characteristics of land cover areas.** Seasonal variation of surface coverage areas in a basin. (a) Line chart showing monthly dynamic changes in the area (km²) of Water, Vegetation, and Mud and Sand throughout a year. (b) Stacked bar chart presenting the monthly proportion (%) of Water, Vegetation, and Mud and Sand. These visualizations illustrate the seasonal changing patterns of different surface types in the basin.

S1 **Fig 10. Frequency Distribution of Water Bodies and Vegetation from 2013 to 2021.** Distribution frequency of water and vegetation in a basin. High - resolution maps showing the distribution frequency of water (a) and vegetation (b) in the basin. For water distribution frequency (a), the legend indicates None, Very Low, Low, Medium, and High levels. For vegetation distribution frequency (b), the legend also marks None, Very Low, Low, Medium, and High levels. These maps illustrate the spatial patterns of water and vegetation occurrence in the basin.

S1 **Fig 11. Stability distribution statistics of water bodies and vegetation from 2013 to 2021.**

Spatial distribution and area statistics of water and vegetation stability in a basin. High - resolution maps showing the spatial distribution of water stability (a) and vegetation stability (b) in the basin, with legends indicating stability levels (Very Low, Low, Medium, High, Very High). Below each map, bar charts present the area (km²) and percentage of each stability level. These visualizations illustrate the stability patterns and quantitative characteristics of water and vegetation in the basin.

S1 Data.Fig 7(b) data:

| Year | Water | Vegetation | Mud and Sand |
| --- | --- | --- | --- |
| 2013 | 51% | 33% | 15% |
| 2014 | 67% | 25% | 8% |
| 2015 | 53% | 29% | 17% |
| 2016 | 54% | 25% | 21% |
| 2017 | 63% | 22% | 15% |
| 2018 | 50% | 32% | 18% |
| 2019 | 65% | 20% | 14% |
| 2020 | 77% | 15% | 8% |
| 2021 | 70% | 19% | 11% |
| 2022 | 46% | 27% | 27% |
| 2023 | 30% | 38% | 32% |
| 2024 | 80% | 9% | 11% |

S1 Data. Fig 9(a) data:

| Type | Jan | Feb | Mar | Apr | May | Jun | July | Aug | Sept | Oct | Nov | Dec |
| --- | --- | --- | --- | --- | --- | --- | --- | --- | --- | --- | --- | --- |
| Water  (km2) | 1417.38 | 1025.12 | 1562.72 | 1775.24 | 2103.11 | 2957.01 | 3020.86 | 2826.17 | 2006.90 | 1615.94 | 1289.44 | 1202.31 |
| Vegetation  (km2) | 1115.44 | 1055.02 | 897.35 | 1069.12 | 1007.67 | 175.66 | 275.48 | 367.41 | 813.21 | 1184.67 | 1293.27 | 1183.53 |
| Mud and Sand(km2) | 761.54 | 1214.22 | 921.39 | 624.16 | 314.36 | 161.69 | 85.21 | 187.94 | 605.02 | 598.37 | 842.42 | 908.52 |

S1 Data. Fig 9(b) data:

| Type | Jan | Feb | Mar | Apr | May | Jun | July | Aug | Sept | Oct | Nov | Dec |
| --- | --- | --- | --- | --- | --- | --- | --- | --- | --- | --- | --- | --- |
| Water | 43% | 31% | 46% | 51% | 61% | 90% | 89% | 84% | 59% | 48% | 38% | 36% |
| Vegetation | 34% | 32% | 27% | 31% | 29% | 5% | 8% | 11% | 24% | 35% | 38% | 36% |
| Mud and Sand | 23% | 37% | 27% | 18% | 9% | 5% | 3% | 6% | 18% | 18% | 25% | 28% |

S1 Data. Fig 11(a) data:

|  | Very Low | Low | Medium | High | Very High |
| --- | --- | --- | --- | --- | --- |
| Area(km²) | 576.47 | 724.53 | 1172.77 | 358.83 | 461.76 |
| Percentage | 17.50% | 21.99% | 35.60% | 10.89% | 14.02% |

S1 Data. Fig 11(b) data:

|  | None | Very Low | Low | Medium | High | Very High |
| --- | --- | --- | --- | --- | --- | --- |
| Area(km²) | 943.38 | 580.35 | 699.65 | 576.83 | 426.96 | 67.20 |
| Percentage | 28.64% | 17.62% | 21.24% | 17.51% | 12.96% | 2.04% |
